# Infections in Infants during the First 12 Months of Life: Role of Placental Malaria and Environmental Factors

**DOI:** 10.1371/journal.pone.0027516

**Published:** 2011-11-11

**Authors:** Agnès Le Port, Laurence Watier, Gilles Cottrell, Smaila Ouédraogo, Célia Dechavanne, Charlotte Pierrat, Antoine Rachas, Julie Bouscaillou, Aziz Bouraima, Achille Massougbodji, Benjamin Fayomi, Anne Thiébaut, Fabrice Chandre, Florence Migot-Nabias, Yves Martin-Prevel, André Garcia, Michel Cot

**Affiliations:** 1 UMR216 Mère et enfant face aux infections tropicales, Institut de Recherche pour le Développement (IRD), Paris, France; 2 Faculté de Pharmacie, Université Paris Descartes, Paris, France; 3 U657, Inserm, Garches, France; 4 EA4499, Université Versailles Saint Quentin (UVSQ), Garches, France; 5 PhEMI, Institut Pasteur, Garches, France; 6 UMR216 Mère et enfant face aux infections tropicales, Institut de Recherche pour le Développement (IRD), Cotonou, Bénin; 7 Institut des Sciences Biomédicales Appliquées (ISBA), Cotonou, Benin; 8 Faculté des Sciences de la Santé (FSS), Cotonou, Benin; 9 UR016 Caractérisation et Contrôle des Populations de Vecteurs, IRD, Montpellier, France; 10 UMR204 Prévention des malnutritions et des pathologies associées (NUTRIPASS), IRD, Montpellier, France; Université Pierre et Marie Curie, France

## Abstract

**Background:**

The association between placental malaria (PM) and first peripheral parasitaemias in early infancy was assessed in Tori Bossito, a rural area of Benin with a careful attention on transmission factors at an individual level.

**Methodology:**

Statistical analysis was performed on 550 infants followed weekly from birth to 12 months. Malaria transmission was assessed by anopheles human landing catches every 6 weeks in 36 sampling houses and season defined by rainfall. Each child was located by GPS and assigned to the closest anopheles sampling house. Data were analysed by survival Cox models, stratified on the possession of insecticide-treated mosquito nets (ITNs) at enrolment.

**Principal Findings:**

Among infants sleeping in a house with an ITN, PM was found to be highly associated to first malaria infections, after adjusting on season, number of anopheles, antenatal care (ANC) visits and maternal severe anaemia. Infants born from a malaria infected placenta had a 2.13 fold increased risk to present a first malaria infection than those born from a non infected placenta ([1.24–3.67], p<0.01) when sleeping in a house with an ITN. The risk to present a first malaria infection was increased by 3.2 to 6.5, according to the level of anopheles exposure (moderate or high levels, compared to the absence of anopheles).

**Conclusions:**

First malaria infections in early childhood can be attributed simultaneously to both PM and high levels of exposure to infected anopheles. Protective measures as Intermittent Preventive Treatment during pregnancy (IPTp) and ITNs, targeted on both mothers and infants should be reinforced, as well as the research on new drugs and insecticides. In parallel, investigations on placental malaria have to be strengthened to better understand the mechanisms involved, and thus to protect adequately the infants high risk group.

## Introduction

Factors influencing the time to first malaria infections in infancy were first investigated by Le Hesran et al. in 1997, who found an effect of placental infection by *P. falciparum* during pregnancy [1]. Two recent studies confirmed the role of placental malaria [2], [3] and were consistent in showing that infants born with a placental malaria (PM) infection were more likely to present a first parasitemia earlier than infants with no history of PM. As an explanation, it was suggested that infant susceptibility to malaria could be influenced by the contact with parasite antigens during in utero life, probably inducing an immune tolerance [1], [4]. However, other factors such as the intensity of malaria transmission and the nutritional status of the child may play a role as well and cannot be ruled out [5], [6], [7]. A multidisciplinary study was set up in Benin, which aimed at determining as completely as possible the parts played by malaria transmission and other biological determinants in first malaria infections, focusing on the role of placental infection and exposure to malaria vectors.

## Methods

### Study population

The study was conducted in the district of Tori Bossito located 40 km North-East of Cotonou, South Benin. The study area included 9 villages and 3 health centres (Tori Avame, Tori Cada and Tori Gare) providing birth attendance and primary health care. A birth cohort was set up in June 2007. Recruitment was performed until July 2008. All women living in any of the nine villages, attending health centres for antenatal care and having no intention to move were proposed to enter in the study. Two months before the study started, study supervisors and community health workers visited all villages to meet the women and to inform them about the study. In addition, midwives were told to present the study to all women attending ANC visits from the 7^th^ month of pregnancy. On arrival at the maternity clinic for delivery, women were again given information on the study protocol. The informed consent written in French and in Fon was then submitted for approval. After delivery, women having had a stillbirth were excluded from the study.

### Baseline Data

For each delivered woman, personal data (age, scholarship), use of Intermittent Preventive Treatment (IPTp) with Sulfadoxine-Pyrimethamine (SP) by the mother and obstetric history were asked at recruitment. Information on the possession of an insecticide-treated net (ITN) by the family at enrolment was collected, according to mother's declaration. A nutritional, entomological, climatological and biological follow-up was then started. At delivery, maternal blood and umbilical cord blood samples were taken to search for malaria infection and anaemia. Thick and thin placental smears were made to look for PM. At birth anthropometric measurements were performed (weight, length, head circumference and mid-upper arm circumference (MUAC)) with methods recommended by the WHO [8].

### Surveillance

**Figure 1:**
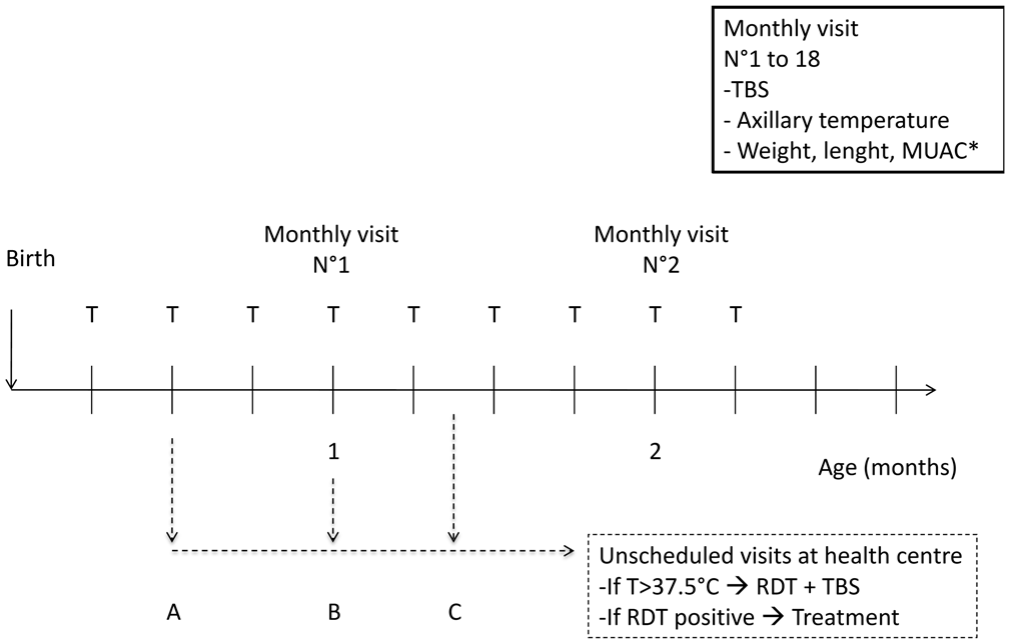
Follow-up diagram of infants from the cohort of Tori Bossito, Benin. 2007–2010. At birth: TBS from mother's blood, placenta and cord blood, anthropometrical measures for new-born; T°: weekly axillary temperature measured by community health workers at infants home; * Anthropometrical measures were done monthly from 1 to 6 months, then at 9, 12, 15 and 18 months; case A (example of unscheduled visits): mother was told during the visit of the community health worker to bring her infant at the health centre because of infant fever or illness (active follow-up); case B: in case of fever or suspected illness of the infant, mother was told during the monthly visit of supervisor and community health worker to bring her infant at the health centre (active follow-up); case C: mother decided to go on her own to the health centre with her infant because of symptoms (passive follow-up); NB: Every trimester, blood samples were taken for haemoglobin measures and immunological assays.

The active follow-up of the infants included several visits (weekly and monthly home visits) from birth up to 18 months (Figure 1). During weekly home visits, axillary temperature was measured by community health workers with a digital thermometer to detect fever. No systematic TBS were performed. Only in case of a temperature higher than 37.5°C, mothers were told to bring their children to the health centre. On arrival at the health centre, a questionnaire was filled up and a Parascreen® rapid diagnostic test (RDT) was made, to obtain an immediate diagnosis of symptomatic malaria infection (i.e. presence of parasites and temperature higher than 37.5°C) for treatment purpose. A thick blood smear (TBS) was performed to provide a later confirmation of the RDT result (up to a few weeks due to work load of the lab technicians). Following a positive RDT, the infant was treated by an artemisinin-based combination (arthemeter and lumefantrine) as recommended by the Beninese National Malaria Control Program.

Monthly visits included a systematic TBS to assess asymptomatic *P. falciparum* infection. Anthropometric measurements (weight, length and MUAC) were performed every month from birth to 6 months, then at 9, 12, 15 and 18 months.

Mothers were also invited to bring their infants to the health centre at any time (passive follow-up), for free attendance in case of fever suspected by the mother or any clinical signs and then the same procedure (i.e. questionnaire and RDT, later confirmed by TBS) was applied. The overall prevalence of HIV is low (2%) in this area [9]. In Tori Bossito, the National AIDS Control Program (NACP) began its activities in 2007. Nine percent (57/617) of all women of the study were tested for HIV infection and only one was found positive. Considering the overall low HIV prevalence, we decided not to take into account these data.

The protocol was approved by both the Beninese Ethical Committee of the Faculté des Sciences de la Santé (FSS) and the IRD Consultative Committee on Professional Conduct and Ethics (CCDE).

### Entomological and environmental follow-up

Entomological data were collected during 2 years, using human landing catches performed every 6 weeks, 3 consecutive nights, in 36 sampling houses (4 houses per village, outdoor and indoor). To account for environmental factors which could influence transmission, climatic parameters (temperature, humidity rate) were recorded and rainfall was measured daily in each village. All infants were located by GPS and assigned to the closest anopheles sampling houses. The distance between infants and catching houses ranged between 0 and 3.1 km with a median at 373 m. Number of anopheles collected in sampling houses was thus available for each infant every 6 weeks.

### Biological and laboratory procedures

All blood samples (maternal blood, cord blood, infant blood) performed on EDTA and placental smears were processed in Tori Bossito laboratory. Malaria is perennial in the study area and according to a recent entomological survey *Plasmodium falciparum* is the commonest species (95%), *P. malariae* and *P. ovale* representing respectively 3% and 2% [10]. During the follow-up, very few TBS were found infected with *P. malariae* and parasitological results are given as Plasmodium sp. TBS performed on placental, maternal and cord blood at delivery were read within 24 hours. TBS performed on the occasion of monthly infant follow-up or during unscheduled consultations were not examined immediately. They were read twice several months later by two lab technicians in the Cotonou laboratory (with less than 1% of disagreement). They were made in the purpose of later confirmation of RDT results. All TBS were stained with Giemsa. Leukocytes and parasites were counted simultaneously. A TBS was declared negative if no parasite was found after 500 leucocytes had been counted. *P. falciparum* asexual blood forms were counted on Giemsa-stained smears. Haemoglobin rates were measured at birth and quarterly on blood samples with a Hemocue® analyser.

### Statistical analysis

As all previous studies had shown an effect of placental infection during the first year of life, and as a full set of data was available only for the first 12 months of follow-up, we restricted our analysis to this period. First, baseline characteristics were compared between infants presenting at least one malaria infection vs no infection during the first year of life, using Pearson chi2 test or Fisher's exact test for categorical variables and Student t-test for quantitative variables. Then several survival analyses were performed.

#### Primary outcome

For survival analyse, first malaria infection (symptomatic or asymptomatic malaria) was defined as the first positive TBS, performed either at monthly home visits or when the child was brought to the health centre because of fever. Such consultations at the health centre could follow a weekly visit (active follow-up) or happen at any time if the mother suspected her child to be sick (passive follow-up).

#### Covariates

Two time-varying covariates were created to take into account factors influencing the exposure to malaria and its fluctuation over time: the season, defined as rainy or dry, according to the start and the end of the rains determined from rainfall measurements, and the number of anopheles collected in the GPS-located sampling houses, coded in 3 classes: 0, [1]–[10], >10 caught anopheles. Spatial covariates (villages or maternity) used in the first univariate analysis (Table S1) were not entered in the multivariate Cox model simultaneously to season and anopheles covariates to avoid redundancy. For the nutritional status, we chose to use the average on 12 months weight-for-age z-score (MWAZ) because anthropometric data were not available every month. It was considered as a fixed and quantitative covariate.

#### Survival analysis

To assess the effect of PM on the time to first infection, Kaplan Meier curves and a log rank test were performed. Univariate Cox analysis was performed to study the association between all covariates and the first malaria infection and to obtain unadjusted hazard ratios (HR). The date of right censoring was the date of first malaria infection or the last available date of follow-up. Then, a multivariate analysis was performed to study the association between first malaria infection (symptomatic or asymptomatic malaria) and PM, adjusting on environmental and other covariates.

Respect of proportional hazard assumption was assessed for all covariates by graphical methods (-Ln(Ln(S)) function of time) and by testing the interaction with time.

Data were analysed with Stata v. 8.0 (Stata Corporation, College Station, Texas, United States) and SAS version 9.0 software (SAS Institute, Cary, North Carolina, United States).

## Results

Data presented in this paper were collected from children recruited between June 4, 2007 and July 31, 2008 and followed during their 12 first months of life. Environmental data (climatic and entomological data) used in this analysis were collected during 2 years, from June 2007 until August 2009.

### Study population

**Figure 2:**
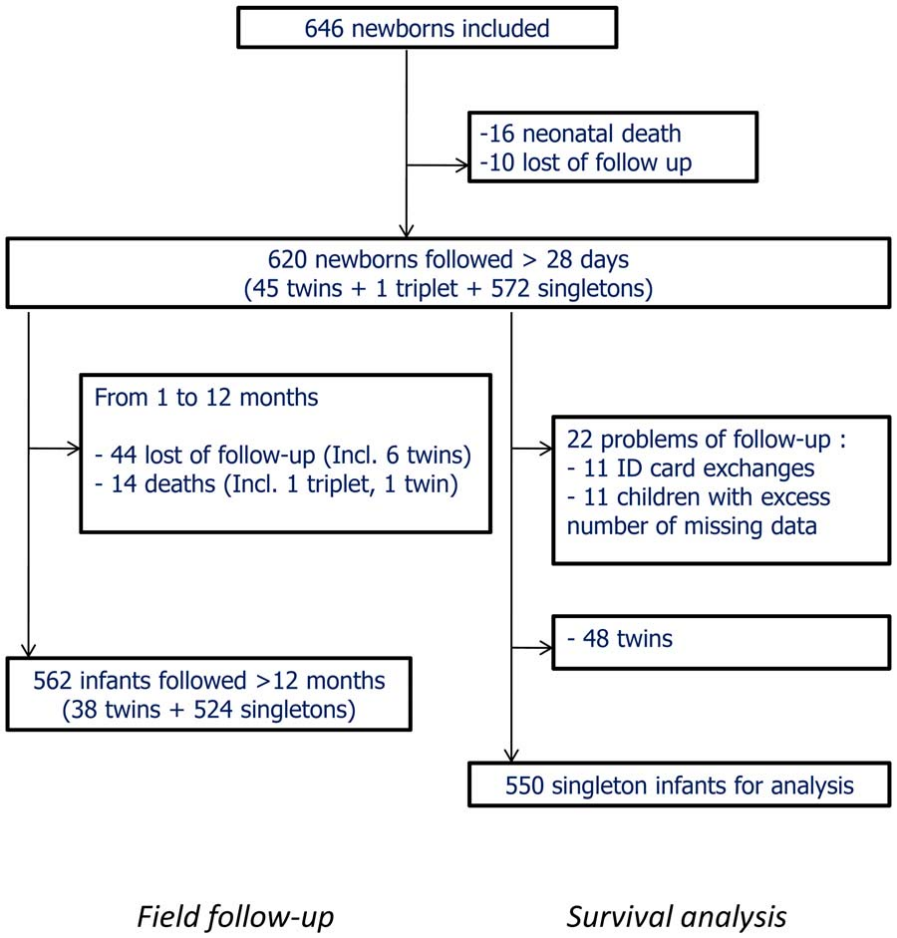
Study flow diagram at 12 months, Tori Bossito study, Benin. Lost of follow-up are children who did not complete the 12-months follow-up (mother's refusal to attend monthly visits). They were considered as censored observations at the time of last visit in survival analysis.

As presented in the flowchart diagram in Figure 2, after exclusion of 10 stillbirths, 646 newborns were included and 620 infants were followed more than one month. Thirty deaths occurred during the 12 months of follow-up and all were investigated. None appeared to be related to malaria complications. Fifty-four infants did not complete the 12 months follow-up, mainly because they moved out of the study area. They were considered as censored observations at the time of last visit in survival analysis. Besides, twenty-two infants out of 620 were excluded from the analysis because of a lower quality of follow-up (more visits missed than performed or doubtful identification). Finally, five hundred fifty live-birth singletons were analysed in survival analysis. The median duration of follow-up was 6 months (ranging from 1 to 12, lower quartile at 3 and upper quartile at 9 months). Neither the 11 children with an excess number of missed visits, nor the overall 22 excluded, differed from the 550 analysed, for the following variables: mother's gravidity, PM, LBW, gender, village of residence and maternity clinic.

### Entomological inoculation rates

The Annual *PfEIR* calculated on the two years duration of the study on all 9 villages indicates the existence of a slight but constant transmission in the area with an average of 20.5 infected anopheles/man/year and two peaks during the rainy seasons, consistent with another study performed in the area [11].

### Malaria infections

One hundred ninety-two infants had at least one episode of malaria infection. Thirty four percent (66/192) of malaria infections were detected during monthly visits and 66% (126/192) during unscheduled visits at the health facility for fever or history of fever. Among monthly visits, 12.1% (8/66) of the infants were feverish. Among unscheduled visits, 96.7% (119/123) of the infants were feverish and 76.4% (94/123) had a history of fever over the past 48 h.

Factors associated with the occurrence of a first parasitaemia during the 12 first months of follow-up are presented in Table S1. In univariate analysis, among infants presenting a first malaria infection, 14.7% were born from a PM versus 8.8% from a non infected placenta (p = 0.03).

Six placental TBS were not available (not performed by midwives or laboratory mishandlings). All covariates directly or indirectly related to exposure such as spatial factors were strongly associated to higher proportions of infants with at least one infection. Proportions varied according to villages (p<0.001) and maternity clinics (p<0.001). Neither age nor gravidity was associated to first malaria infections, whereas malaria prevention measures (IPTp, ITNs) or the attendance to more than 3 antenatal care visits had a protective effect.

Characteristics of infants had no effect on proportions of children presenting at least one malaria infection (gender, LBW, MWAZ).

### Congenital infections

TBS on cord blood was positive in 5 cases out of 604 samples (0.83%). These 5 infants were controlled at day 1 and no parasite was found in the peripheral blood TBS. Among these 5 infants, all were born from PM infected mothers and two of them further had a first malaria infection at one month and three months of age, whereas the others presented no detectable infection until the age of 12 months.

### Kaplan-Meier analysis

**Figure 3:**
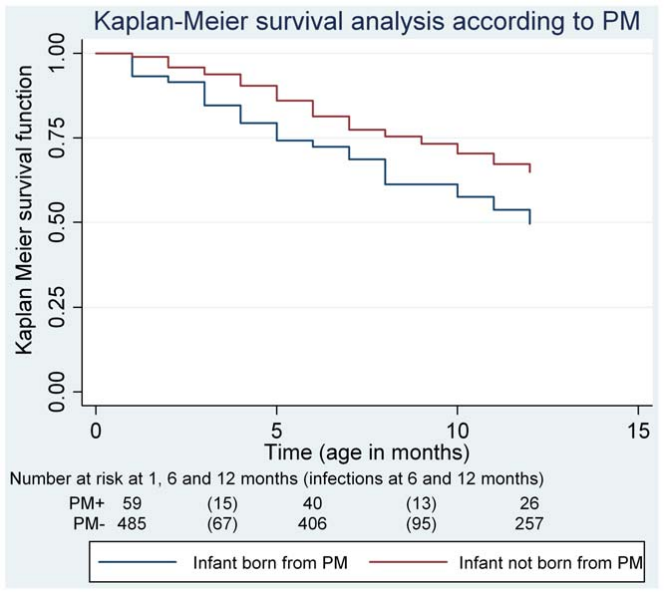
Kaplan-Meier probability of 12-month occurrence of malaria infection in 550 infants followed from birth in Tori Bossito, Benin, 2007–2009.

The median time (range) of first malaria infection was 4.88 months (0.53–11.86) in infants born from infected placentas, while it was 6.11 months (0.53–12.15) in infants born from non infected placentas. Four point seven percent (9/192) of the infants presented a first infection at one month, 20.3% (39/192) before 3 months and 54.7% (105/192) before six months of age. In all time classes, these proportions were lower in the children with an ITN at home compared with the children without ITN (data not shown). For a better understanding of the occurrence of malaria in relation to the possession of an ITN, we calculated the proportions of infants who presented a first malaria infection according to PM infection and ITN possession (Table 1). Among the owners of an ITN, there were less PM- than PM+ children infected in the first year. We also calculated the median [min-max] time (in months) to first infection for infants with and without ITNs in relation to PM status (Table 2). The Kaplan Meier curves, according to PM, are presented in Figure 3. Infant survival probabilities (not to present a first malaria infection) at 6 months were respectively 0.74 (95%CI [0.61–0.83]) and 0.84 (95%CI [0.80–0.87]) according to the existence of PM or not and 0.49 (95%CI [0.35–0.62]) and 0.64 (95%CI [0.60–0.69]) at 12 months. Infants born from infected placentas had a higher probability to present a first malaria infection than infants born from non infected placenta (Log-Rank test, p = 0.01).

**Table 1:** Proportions of infants with first malaria infection, according to PM infection and ITN possession at enrolment, chi^2^ test.

		PM- (n, %)	PM+ (n, %)	
No possession of ITN				
	No infection	92 (58.23)	14 (56.00)	p = 0.83
	At least one infection	66 (41.77)	11 (44.00)	
ITN possession				
	No infection	229 (71.34)	17 (50.00)	p = 0.01
	At least one infection	92 (28.66)	17 (50.00)	

**Table 2:** Median (range) time to first infection for infants with and without ITN and from PM infected mother or not (in months).

	PM-	PM+
No possession of ITN	5.42, [0.53–12.16], (n = 66)	3.35, [0.85–10.84], (n = 11)
ITN possession	6.52, [1.02–11.89], (n = 92)	7.10, [0.52–11.86], (n = 17)

### Cox univariate analysis

Results are presented in Table 3. While an older age of the mother, and a high number of ANC visits (>3) were associated with a lower risk of first infections, the existence of a PM at delivery was associated with a higher risk. Infants born from an infected placenta had an HR = 1.62 [1.08–2.43, p = 0.02] to present a first malaria infection compared to infants without a history of PM. High exposures to environmental factors (anopheles and season) were also strongly associated with a higher risk of first malaria infection whereas the possession of ITNs at the beginning of the follow-up or the intake of IPTp by the mothers were associated with a lower risk of infection. Spatial factors as villages or maternity location were highly associated to first infections. Interestingly, neither the gravidity status of the mother nor any of the infants' characteristics (gender, LBW, MWAZ) were associated with a risk of malaria infection in the 12 months of follow-up.

**Table 3:** Factors associated with first malaria infection by Cox univariate analysis.

			Unadjusted HR (95% CI)	p
Maternal factors				
	Age class			
		≤20	1	
		21–25	0.89 [0.61–1.30]	p = 0.55
		26–30	0.66 [0.44–0.98]	p = 0.04
		>30	0.65 [0.43–0.98]	p = 0.04
	Placental malaria			
		no	1	
		yes	1.62 [1.08–2.43]	p = 0.02
	Maternal anaemia (<7g/dl)			
		no	1	
		yes	2.64 [0.98–7.13]	p = 0.05
	Gravidity status			
		Multigravidity	1	
		Primigravidity	1.11 [0.75–1.63]	p = 0.59
	Bed net possession			
		no	1	
		yes	0.64 [0.48–0.86]	p = 0.001
	IPTp use			
		no	1	
		yes	0.65 [0.46–0.92]	p = 0.01
	Number of ANC			
		< = 3 ANC	1	
		>3 ANC	0.68 [0.51–0.92]	p = 0.01
	Education of women			
		No education	1	
		Partial primary	0.64 [0.37–1.12]	p = 0.12
		Complete primary or more	2.26 [1.31–3.92]	p<0.001
Infants factors				
	Gender			
		Female	1	p = 0.77
		Male	1.04 [0.78–1.39]	
	LBW			
		no	1	p = 0.73
		yes	0.92 [0.56–1.51]	
	Mean weight-for-age z-score (MWAZ)		0.89 [0.77–1.03]	p = 0.13
				
Environmental factor (Time-varying covariates)				
	Anopheles exposure			
		0	1	
		[1–10[	2.82 [1.99–3.99]	p<0.001
		[>10[	4.68 [3.00–7.29]	p<0.001
	Season			
		Dry	1	
		Rainy	1.98 [1.18–3.30]	p<0.001

Tori Bossito, Benin. 2007–2009.

### Cox multivariate analysis

All covariates respected the proportional hazard assumption, except the ITN covariate. Graphical methods showed that risks were not proportional with time and an interaction between ITN and age was found statistically significant (p = 0.04). Therefore, we decided to perform two separate analyses, according to the possession of an ITN or not. Results of the two analyses are presented in Table 4.

**Table 4:** Factors associated with first malaria infection according to possession of ITNs by Cox multivariate analysis.

			Possession of ITNs (n = 361)*		No possession of ITNs (n = 183)	
			Adjusted HR[95% CI]	p	Adjusted HR[95% CI]	p
Maternal factors						
	Placental malaria					
		no	1		1	
		yes	2.13 [1.24–3.67]	p<0.01	1.18 [0.60–2.33]	p = 0.62
	Maternal anaemia (<7g/dl)					
		no	1		1	
		yes	5.89 [1.28–27.14]	p = 0.02	1.82 [0.44–7.60]	p = 0.41
	Number of ANC					
		< = 3 ANC	1		1	
		>3 ANC	0.73 [0.49–1.08]	p = 0.11	0.79 [0.49–1.27]	p = 0.33
Environmental factors (Time-varying covariates)						
	Exposure to anopheles					
		0	1		1	
		[1]–[10]	3.20 [1.93–5.31]	p<0.001	1.75 [1.01–3.06]	p = 0.05
		[>10]	6.50 [3.56–11.84]	p<0.01	1.64 [0.71–3.80]	p = 0.25
	Season					
		dry season	1		1	
		rainy season	1.99 [0.96–4.15]	p = 0.06	2.43 [0.95–6.19]	p = 0.06

Tori Bossito, Benin. 2007–2009.

*Women and infants differed significantly according to the possession of ITNs in proportion of primigravidae (28.4% within houses with no ITNs vs 8.6% within houses with ITNs, p<0.001), proportion of women taking IPTp (79.8% vs 86.4% respectively, p<0.05), attending more than 3 ANC visits (48.8% vs 60.7%, p = 0.01) and in proportion of LBW (16.5% vs 5.5%, p<0.001).

In the first group (i.e. possessing an ITN, N = 361), the role of PM remained strongly significant in increasing the risk of a subsequent first malaria infection, after adjustment on exposure to anopheles, season, ANC visits and severe maternal anaemia. Infants with a history of PM had a 2.1 fold increased risk to present a first malaria infection compared to infants with no history of PM ([1.24–3.67], p<0.01).

Infants exposed to a high number of anopheles (>10 anopheles/3 days-collection) had a HR = 6.50, [3.56–11.84], p<0.001 to present a first malaria infection compared to infants not exposed to anopheles, at a given age. There was an increasing HR with the amount of exposure to anopheles, as the infants exposed to a low number of anopheles (1-10 anopheles/3 days-collection) had a HR = 3.20, [1.93–5.31], p<0.001 to present a first malaria infection compared to infants not exposed to anopheles.

In the second group (i.e. not possessing an ITN (N = 183), first malaria infections were not statistically associated with PM at delivery with an HR = 1.18, [0.60–2.33], p = 0.62 and were borderline associated with an exposure to a low number of anopheles (p = 0.05).

First malaria infections were borderline associated to the season, among infants in both groups (p = 0.06).

## Discussion

The main result of our study is that first malaria infections in infants are associated with PM during pregnancy, and this effect persists after adjustment on environmental factors related to malaria transmission such as the exposure to anopheles bites or the season. It confirms the results of previous studies, while eliminating the possibility that PM could be only a proxy variable for malaria transmission. Indeed, the most logical alternative explanation for early infections in infants would be a massive exposure to malaria vectors, testified by a high rate of PM infections during the mothers' pregnancies.

In our study, infants born from infected placentas (whatever the presence of ITNs in their homes) had a 1.6 fold increased risk to present a first infection than infants born from non infected placentas (p = 0.02). This result is consistent with the three main studies conducted previously in different parts of Africa, in Cameroon (1997), Tanzania (2005) and Gabon (2008) [1], [2], [3]. However in these studies, measures of exposure to malaria vectors at an individual level had not been accounted for simultaneously and an uncertainty remained on the respective effects of placental malaria and vector transmission. In this respect, our findings may provide a final answer to the debate (see Cairns and Luty [5]), as we found that both PM and transmission played a role in reducing the time to first malaria infections.

In the survival analyses, a significant relation was found between the risk of first infection and time-related environmental factors (anopheles catches and season). There was also a significant association between PM and the risk of first infection, particularly pronounced in infants whose mothers had declared to possess an ITN (Cox model), after adjusting for environmental covariates. The same association was not found in the non-ITN strata. The effect of ITNs on malaria transmission is certainly important. A Cochrane meta-analysis concerning areas of stable transmission (i.e. EIR>10 infective anopheles bites/man/year) comparable to Tori Bossito, estimated at 50% the ITN-attributable reduction in clinical episodes, and 13% the reduction of infection prevalence in children[12]. In this respect, an explanation to our results could be a dramatic reduction in transmission provided by the use of ITNs, allowing the effect of PM to appear in this group only. In unprotected children, a much higher transmission could “saturate” the individual ability to modulate the occurrence of parasitemias in over-exposed children.

In our study however, the information on ITN possession was collected by interview, and the entomologists involved in the study reported a limited use of ITNs by the population, so this variable should be interpreted with caution. The effect of ITNs deserves to be explored further, looking more carefully to environmental and behavioural factors, over a longer time of observation.

We chose to use the season and the exposure to anopheles as proxies for malaria transmission for two main reasons. First, the seasonality in transmission has been shown to be logically related to the number of clinical malaria cases[13], [14], [15]. Besides, we thought that a number of caught anopheles over time, in a nearby mosquito sampling house would better reflect an individual exposure to vectors than the commonly used EIR which is more globally related to a geographical area. However, a limitation to these sampling methods is that transmission is calculated on the basis of anopheles human catches performed on adults instead of children, which may lead to discrepancies in the estimation of infection rates [16]. In addition, the use of satellite remote sensing has shown a high spatial variability of transmission in various malaria endemic areas [17], [18]. In our study, the estimation of transmission may differ between the sampling house and the child's home. Nevertheless we think that the number of caught anopheles was a rather good indicator of transmission in the target population, as confirmed by our results. Indeed, among infants living in homes with ITNs, those exposed to moderate to high levels of anopheles had a 3.2 to 6.5-fold increased risk to present a first malaria infection compared to infants living near sampling houses where no anopheles were captured.

Concerning the detection of first malaria infections, we had to deal with various sources of information. A parasitaemia could be either found on the occasion of a monthly systematic visit including TBS, or after a weekly household visit by a community health worker, or following the mother's own-decision (supposedly because of a sick child) to attend the health centre. In the two latter cases, the parasitological finding would be guided by clinical signs (mainly fever) in the former a positive TBS could be found either in a symptomatic or asymptomatic context. In order to detect as many “first infections” as possible, we decided to merge all data, whatever their origin. There is a possibility that some asymptomatic parasitaemias could have been missed using such a procedure, but the only alternative way to detect all events would have been an active parasitological weekly follow-up for each child, with the risk of poor acceptance by the community. We chose not to increase the burden of biological samplings in children. Finally, it seems unlikely that an under-detection of some malarial events could have biased our results of association between PM and first parasitaemia. In addition, we think the use of Cox models taking into account the delay to event (first malaria infection or last visit in case of loss to follow-up) instead of the calendar date has improved our statistical analysis.

The absence of PCR testing on the whole cohort is probably a limitation of the study. On cord blood, it would have increased the sensitivity of the detection of congenital infections by 3 to 5-fold, according to previous studies [19], [20], [21]. Further testing of infants during the first three months of life could also have helped to determine the role of congenital infections in the occurrence of parasitaemias, even if the similarity of genotypes between placental and cord blood strains has not been completely established so far [22], [23]. In our study, limited to microscopic examination, among the 5 newborns presenting parasites in the cord blood, only one was found infected in the first month of life. It suggests that congenital malaria infections may not play a prominent role in further infections occurring in children. This hypothesis should be verified by an ongoing sub-study on a limited number of mother-child pairs, tested by PCR (C. Dechavanne, personnal communication).

On placental blood, PCR would have shown submiscroscopic infections, which might also have consequences on birth outcomes [19], [20]. However, we used placental smear as the standard technique. It allows comparisons with previous studies on the occurrence of first infections in children, which did not use molecular diagnosis methods.

Contrary to previous studies [2], [3], we did not evidence any association between the time to first malaria infection and the mother's gravidity. Infants born from primigravidae or multigravidae had similar risks to present a first malaria infection, even after adjustment on PM and other covariates, primigravidae being more at risk to present a PM than multigravidae as usually described. There is no straightforward physiological mechanism to sustain the hypothesis of an increased risk of early infection in children born from multigravidae. Malhotra and coll. who recently investigated the immunological mechanisms underlying infant susceptibility to malaria, found no evidence of an effect of maternal parity on the risk of subsequent infection during early childhood either [24].

Finally, first malaria infection in early childhood can be attributed simultaneously to both PM and high levels of exposure to infected anopheles. It emphasizes the importance of gestational malaria as a threat to the child's health, by increasing the risk of low birth weight, but also per se, by inducing a susceptibility to further malaria attacks. Protective measures as IPTp and ITNs, targeted on both mothers and infants should be reinforced, as stated by the Roll Back Malaria initiative in the Abudja declaration [25]. In parallel, investigations on placental malaria should be continued to understand the mechanisms involved, as conditions could rapidly change with the appearance of anopheles resistance to insecticides or the quick increase of parasite resistance to SP.

## Supporting Information

Table S1 Baseline characteristics of infants with ≥1 malaria infection during the first 12 months of follow-up, Tori Bossito, Benin, 2007–2010. * Student t-test (equal variances in the two groups). £ Six placental malaria TBS were missing.
(DOCX)
