# Supplementary material for: Infections in Infants during the First 12 Months of Life: Role of Placental Malaria and Environmental Factors
Source: PLoS One. 2011 Nov 11;6(11):e27516. doi: 10.1371/journal.pone.0027516 (PMC3214070; doi:10.1371/journal.pone.0027516)
Supplement: Table S1 — Baseline characteristics of infants with ≥1 malaria infection during the first 12 months of follow-up, Tori Bossito, Benin, 2007–2010. * Student t-test (equal variances in the two groups). £ Six placental malaria TBS were missing. (DOCX) [file pone.0027516.s001.docx]

|  |  |  | **Infants with ≥1 malaria infection (n,%)** | **Infants with no malaria infection (n,%)** | **Total (n)** | **p** |
| --- | --- | --- | --- | --- | --- | --- |
| **Maternal factors** |  |  |  |  |  |  |
|  | Age class |  |  |  |  |  |
|  |  | ≤20 | 28(14.58) | 48 (13.48) | 76 | p=0.12 |
|  |  | 21-25 | 56 (29.17) | 75 (21.07) | 131 |  |
|  |  | 26-30 | 64 (33.33) | 128 (35.96) | 192 |  |
|  |  | >30 | 44 (22.92) | 105 (29.33) | 149 |  |
|  | Placental malaria £ |  |  |  |  |  |
|  |  | no | 162 (85.26) | 323 (91.24) | 485 | p=0.03£ |
|  |  | yes | 28 (14.74) | 31 (8.76) | 59 |  |
|  | Gravidity status |  |  |  |  |  |
|  |  | Multigravidity | 161 (83.85) | 306 (85.47) | 467 | p=0.61 |
|  |  | Primigravidity | 31 (16.15) | 52 (14.52) | 83 |  |
|  | Bed net possession |  |  |  |  |  |
|  |  | no | 77 (40.96) | 106 (29.78) | 183 | p<0.01 |
|  |  | yes | 111 (59.04) | 250 (70.22) | 361 |  |
|  | IPTp use |  |  |  |  |  |
|  |  | no | 41 (21.58) | 48 (13.45) | 89 | p=0.01 |
|  |  | yes | 149 (78.42) | 309 (86.55) | 458 |  |
|  | Number of ANC |  |  |  |  |  |
|  |  | <= 3 ANC | 92 (50.55) | 136 (39.65) | 228 | p=0.02 |
|  |  | >3 ANC | 90 (49.45) | 207 (60.35) | 297 |  |
|  | Education of women |  |  |  |  |  |
|  |  | No education | 164 (85.42) | 303 (84.64) | 467 | p=0.03 |
|  |  | Partial primary | 14 (7.29) | 43 (12.01) | 57 |  |
|  |  | Complete primary or more | 14 (7.29) | 12 (3.35) | 26 |  |
|  | Maternal anaemia (<7g/dl) |  |  |  |  |  |
|  |  | no | 185 (97.89) | 350 (99.43) | 535 | p=0.09 |
|  |  | yes | 4 (2.11) | 2 (0.57) | 6 |  |
| **Infants factors** |  |  |  |  |  |  |
|  | Gender |  |  |  |  |  |
|  |  | Female | 94 (49.21) | 180 (50.56) | 274 | p=0.76 |
|  |  | Male | 97 (50.79) | 176 (49.44) | 273 |  |
|  | LBW |  |  |  |  |  |
|  |  | no | 175 (91.15) | 323 (90.48) | 498 | p=0.80 |
|  |  | yes | 17 (8.85) | 34 (9.52) | 51 |  |
|  | Mean weight-for-age  z-score (MWAZ) * |  | - 0.8274 (SD=0.07; min=-3.71; max=1.87) | -0.6828 (SD=0.05;min=-4.91; max=2.84) |  | p=0.11 |
| **Location factor** |  |  |  |  |  |  |
|  | Village (anopheles catches) |  |  |  |  |  |
|  |  | Avame centre | 15 (7.81) | 73 (20.39) | 88 | p=0.001 |
|  |  | Gbedjougo | 8 (4.17) | 71 (19.83) | 79 |  |
|  |  | Houngo | 5 (2.60) | 14 (3.91) | 19 |  |
|  |  | Ananvie | 28 (14.58) | 35 (9.78) | 63 |  |
|  |  | Dohinoko | 24 (12.5) | 59 (16.48) | 83 |  |
|  |  | Gbetaga | 35 (18.23) | 18 (5.03) | 53 |  |
|  |  | Cada centre | 36 (18.75) | 45 (12.57) | 81 |  |
|  |  | Zebe | 12 (6.25) | 27 (7.54) | 39 |  |
|  |  | Zoungoudo | 29 (15.11) | 16 (4.47) | 45 |  |
|  | Maternity of delivery |  |  |  |  |  |
|  |  | Tori Avame | 26 (13.54) | 153 (42.74) | 179 | p=0.001 |
|  |  | Tori Cada | 133 (69.27) | 134 (37.43) | 267 |  |
|  |  | Tori Gare | 33 (17.19) | 71 (19.83) | 104 |  |

Table 1: Baseline characteristics of infants with ≥ 1 malaria infection during the first 12 months of follow-up, Tori Bossito, Benin, 2007-2010

* Student t-test (equal variances in the two groups)

£ Six placental malaria TBS were missing
